# Use of Chemical and Colorimetric Changes to Age *Cryptotermes brevis* Frass for Termite Management

**DOI:** 10.3390/insects15120924

**Published:** 2024-11-26

**Authors:** William Haigh, Babar Hassan, Tengfei Yi, R. Andrew Hayes

**Affiliations:** 1 Forest Research Institute, University of the Sunshine Coast, Sippy Downs 4556, Australia; tyi@usc.edu.au (T.Y.); rhayes@usc.edu.au (R.A.H.); 2 School of Science, Technology and Engineering, University of the Sunshine Coast, Sippy Downs 4556, Australia; 3 Queensland Department of Agriculture and Fisheries, Salisbury 4107, Australia; babar.hassan@daf.qld.gov.au

**Keywords:** West Indian drywood termite, frass chemistry, pest management, fumigation

## Abstract

Drywood termites are problematic timber pests worldwide. *Cryptotermes brevis*, the West Indian drywood termite, is among the most important of these pests, in part due to its cryptic nature, making it hard to detect in structures and thereby easy to transport. Primary detections are commonly made by the observation of frass deposits evacuated through kick-holes, often leading to fumigation of the building to effectively eradicate the pest. However, after treatment, new frass piles are often discovered. It is, then, challenging to determine whether this frass represents reinfestation or is simply old frass dislodged from inactive nests. This may lead to unchecked termite proliferation or expensive fumigation when it is not needed. The ability to assess the age of the found frass would allow inspectors to determine whether a new infestation has occurred. Photocolorimetry has indicated that frass darkens over time, but large differences in the starting color of frass samples limit the usefulness of this as an aging method. Gas chromatographic-mass spectrometric analysis of the hexane extracts of *C. brevis* frass samples aged 0–22 months found differences between fresh frass (under 6 months) and those aged over 12 months but could not distinguish between 6- and 12-month-aged frass. Eight compounds accounted for over 70% of the differences between samples and are, thus, targets for chemical analysis of frass of unknown age. While this chemical frass analysis requires fairly sophisticated analytical tools, the cost associated with these processes would pale in comparison with those for unnecessarily fumigating a structure and thus merits further refinement to increase the accessibility of the method.

## Simple Summary

The West Indian drywood termite, *Cryptotermes brevis*, is one of the most widespread and damaging termite pests in the world. After the successful fumigation of an infested property, evidence of the termite can still be present in the form of feces, or frass. At some time after fumigation, a reliable method for aging this frass would be valuable as new deposits of it may be detected. It is vital to ascertain whether these are from a new infestation or left over from the previous attack. We examined whether the frass changed in color or chemical profile as it aged. Change in color is not a useful tool for such a determination, but the frass does change chemically. This research provides a useful method for determining if frass is old or new and will assist in the management of this invasive pest.

## 1. Introduction

Termites cause massive damage to timber in service across the world. Drywood termites can be especially problematic because they attack wood well out of soil contact and can cause considerable damage before they are detected [1]. The cryptic lifestyles of drywood termites also create a remarkable ability to be transported as a hitchhiker pest on wood products [2], resulting in widespread global distribution. One of the most widespread and destructive drywood termite species is the West Indian drywood termite, *Cryptotermes brevis* (Walker) [3]. This species is invasive in many subtropical regions across the world, including Australia [4]. While precise global estimates are difficult to ascertain, drywood termites are thought to be responsible for around 20% of the total annual expenditure on termite control, which amounts to around USD 40 billion [5]. Many countries report spending millions of dollars annually solely for managing *C. brevis*. The most heavily impacted countries include the United States, Australia, Spain, and South Africa, as well as numerous islands in the Pacific and Atlantic regions [6].

A large part of the impact of these termites comes from attempts to eradicate colonies from structures. Minor drywood termite infestations can be remedied by removing the infested components, though issues arise concerning colonies within load-bearing timbers. Larger infestations are generally eliminated by structural fumigation, commonly using sulfuryl fluoride [7]. Fumigation is generally effective at killing termites but requires the occupants to move out of their homes during treatment and is also costly, especially in countries with fewer established service providers [2]. The cost and inconvenience can prevent individuals from proceeding with fumigation, enabling the termites to propagate within and beyond the initial infestation. Other management solutions for minor infestations include spot treatments utilizing heat or gas/liquid-propelled termiticides [8,9,10]. Spot treatments can largely reduce management costs but crucially require the ability to detect colony locations precisely. Advancements such as radar technologies, among others, show promise for detecting infestations [11]. Importantly, while chemical treatment may leave a residue with some termiticidal effects, the majority of these treatment methods do not prevent reinfestation of the structure from any adjacent infestations [12], leading to increased cost and stress from recurring infestations and exacerbating the impact that *C. brevis* has on a location.

Drywood termite colonies are cryptic, so they are normally detected by the presence of discarded wings from emerging alates or from frass deposits (fecal pellets) in the structure. Frass is commonly discovered in conical piles or is scattered across horizontal surfaces beneath infested wood. These hexagonally shaped pellets serve as a diagnostic feature for drywood termites, providing characteristic identification at the species level [13,14]. However, drywood termites, including *C. brevis*, often accumulate significant quantities of frass within their nests, which can fall out due to external forces or influences, such as ants and other insects inhabiting vacant termite nests and pushing this frass out [15]. These circumstances result in distinct piles that are often mistaken for termite activity, leading to false detection and subsequent costly and unnecessary re-treatments [4]. Distinguishing between old frass from the galleries of inactive colonies and fresh frass from active termite colonies would allow inspectors to determine if further action is needed.

One potential method for identifying recently produced frass could involve observations of the color of frass deposits to date the time since production. Anecdotal evidence suggests that *C. brevis* frass darkens as it ages (Fitzgerald, pers. comm.), and this has been suggested as a possible frass aging method. Alternatively, frass may be chemically characterized and then assessed for changes over time to determine its age as components oxidize or volatilize [16,17,18]. Lewis et al. [19] found that the hydrocarbons in frass from another drywood termite species, *Incisitermes minor*, displayed such a change. A similar trend for *C. brevis* frass could allow samples to be categorized by age to determine if reinfestation has occurred.

This study was conducted to explore the use of color or chemical changes in *C. brevis* frass to determine frass age, with the aim of reducing unnecessary fumigation by identifying frass of pre-fumigation origins and lowering the demand for localized treatment where infestations are no longer present, decreasing the economic burden caused by this pest.

## 2. Materials and Methods

### 2.1. Termite Collection

*Cryptotermes brevis* colonies were collected from an infested building in Maryborough, Queensland, Australia (−25.54, 152.71). The species was confirmed by frass examination, and then infested materials (such as floorboards, doors, and panels) were transported back to the laboratory at the Ecosciences Precinct, Brisbane. The infested material was kept at 25 °C and 75% R.H. before the termites were manually extracted from the wooden material using a chisel. In Australia, *C. brevis* has been found predominantly in structures made from hoop pine (*Araucaria cunninghami*), so this was chosen as a suitable substrate for maintaining the colonies. Collected colony members were maintained on veneers of hoop pine for a minimum of four weeks, to ensure that any other material in their guts had passed through and that any termites damaged during collection were eliminated.

### 2.2. Frass Collection and Ageing

Colonies of acclimated termites (100 members, with 1 male and 1 female reproductive and 98 pseudergates) were then moved into plastic containers containing recently cut hoop pine veneers, taped to form galleries of at least 20 cm^2^ in cross-section. Three replicate colonies were formed with hoop pine veneers of different thicknesses in each group, to allow natural variety to the colony construction between groups. Colonies were left undisturbed in the dark at 25 °C and 75% R.H. Approximately once a month, the containers were opened, and all frass produced by the termites was collected manually using a fine paintbrush. The frass was weighed and stored on open plastic trays, under the same conditions as the termites, from collection until analysis to imitate natural aging. Collections for each proposed method were performed independently. For the color analysis, the frass was collected from each colony, with a portion being analyzed immediately and the remainder left to age prior to re-analysis. For the chemical study, frass was collected approximately monthly over a period of 22 months, with 19 sampling events for each colony.

### 2.3. Frass Color Analysis

**Figure 1:**
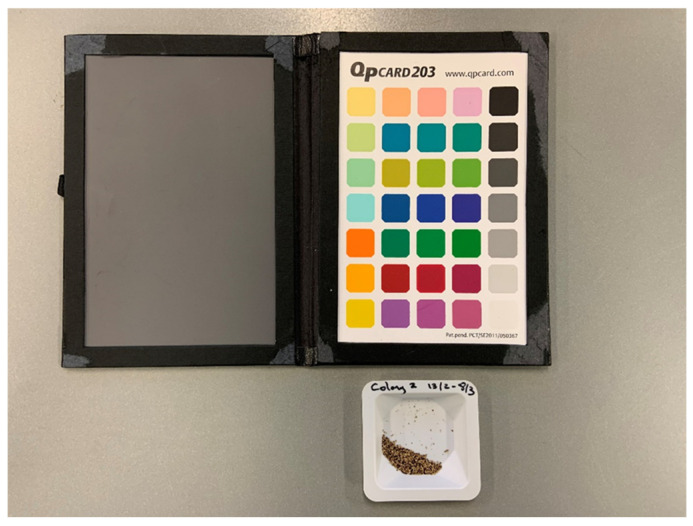
Sample of *C. brevis* frass used to measure L*, a*, and b* in the CIELAB color space and the QP card used to correct for color variations.

Images of the whole frass sample that was collected (128.1 ± 13.2 mg) were taken at the start of experimentation and after aging with an iPhone XR (Apple, Palo Alto, CA, USA) containing a 12 M-pixel (f/1.8) camera using a 9 W LED light source, with the images captured in a closed space from a fixed distance of 20 cm to ensure consistency of images between the imaging events. The resulting images were analyzed for changes in color using ImageJ v.1.52n software (National Institutes of Health, Bethesda, MD, USA). QP card 203 (QPcard AB, Helsingborg, Sweden) was used for the color correction of samples through the “Calibrate” tool of ImageJ (Figure 1). This process was critical for standardizing color changes in the sample surfaces at each time point. The image colors were then divided into their L*, a*, and b* parameters. The color parameter values were obtained from ImageJ by measuring selected areas of each sample automatically.

The CIELAB system was used to characterize any color changes; L* represented lightness (0—black, 100—white), while a* and b* represented chromaticity parameters on the green–red (negative toward green, positive toward red) and blue–yellow (negative toward blue, positive toward yellow) axes, respectively. The overall color change (∆E*) was calculated based on Equation (1):

(1) $$\Delta E_{ab}^{*}=\sqrt{{\left(L_{2}^{*}-L_{1}^{*}\right)}^{2}+{\left(a_{2}^{*}-a_{1}^{*}\right)}^{2}+{\left(b_{2}^{*}-b_{1}^{*}\right)}^{2}}$$

where ΔL*, Δa*, and Δb* represent the differences in the initial and final values of L*, a*, and b*, respectively, and human perception of a “just noticeable difference” (JND) between two colors is defined as ∆E*_ab_ ≈ 2.3 [20]. The data were then used to calculate Euclidian distance values to provide better comparisons between samples that had been aged for different time periods.

### 2.4. Chemical Analysis

After aging the samples, frass samples from each colony and time point were placed into 1 mL hexane and mixed for 24 h on a shaker table (~80 rpm). The solvent was then filtered off, evaporated to dryness under nitrogen gas, and redissolved in hexane (100 µL). A 3 µL sample of the extract was analyzed by gas chromatography-mass spectrometry (GC-MS) (Agilent 6890 and Agilent 5975, respectively). The GC-MS conditions were optimized based on Haverty et al. [21] and were as follows: inlet temperature 250 °C, carrier gas helium at 15 cm s^−1^, split ratio 13:1, transfer-line temperature 280 °C, initial temperature 40 °C, rate 1: 40 °C min^−1^ to 200 °C, rate 2: 3 °C min^−1^ to 300 °C, and final time 11 min. The MS was held at 280 °C in the ion source, with a scan rate of 2.40 scans s^−1^. The resulting peaks were assessed, and identities were tentatively assigned using the National Institute of Standards and Technology (NIST) mass spectral library [22]. The frass collections were arbitrarily categorized as being fresh (0–196 days of storage), medium (197 to 381 days of storage), or old (382 to 690 days of storage) (Table 1). The chemicals detected in each extract were then compared between age classes.

### 2.5. Data Analysis

To determine the differences among age classes for each compound identified, we used a Friedman test to compare the mean ranks, with post hoc analysis by Wilcoxon signed-rank tests. Analyses were performed using IBM SPSS Statistics (V 29.0.0.0). The overall volatile profile of the frass was compared using non-metric multidimensional scaling (nMDS) ordination on a Bray–Curtis similarity index using the relative areas under chromatographic peaks. An analysis of similarity (ANOSIM) was used to compare between age classes, while a similarity percentages (SIMPER) analysis identified the peaks that significantly contributed to any variations between classes. Analyses were performed using PRIMER-e (V 7.0.23).

## 3. Results

### 3.1. Color Analysis

The color values for freshly collected frass varied widely between the three colonies (Table 2), as did the Euclidian distance values for comparing fresh vs. aged frass (Table 3).

**Figure 2:**
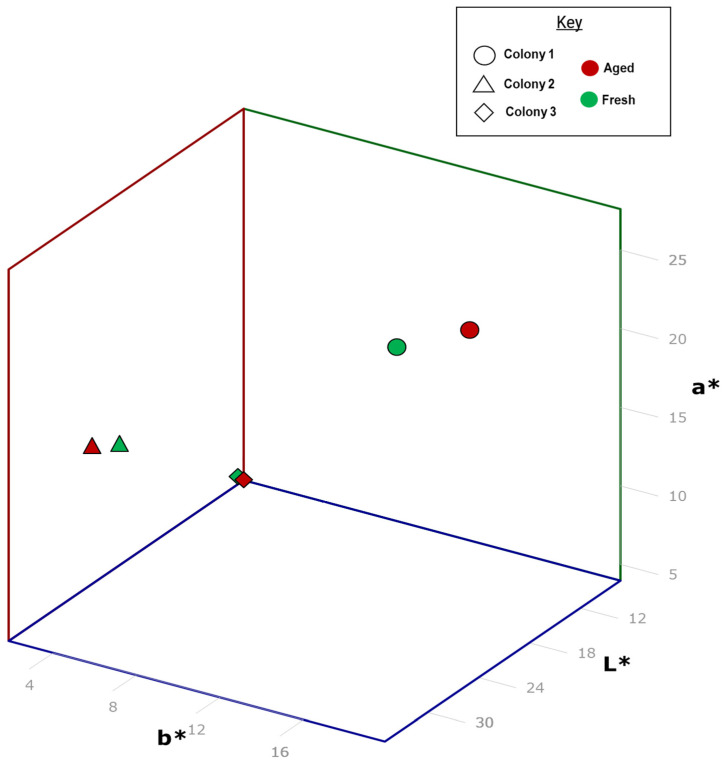
Color changes seen in L*, a*, and b* in *C. brevis* frass as it ages, recorded in the CIELAB color space and mapped to a visual 3-D plot. The axes display the color variable values (0–100 scale). A slight distinction is observed between age groups, though a larger discrepancy is shown between colonies.

For each colony, there was a difference between the fresh and aged frass, and this change was largely driven by a decrease in the L* value for colonies 1 and 3, i.e., the frass sample darkened, with shorter aging time displaying a smaller change and colony 2 displaying very little change due to aging (Table 2). While there were some differences in ∆E* in the frass samples over time, the differences between individual fresh samples were much greater (Figure 2), even when the termites were fed on an identical food source. Thus, color change is unlikely to be a useful tool for assessing frass age without having samples taken at time zero to provide a baseline for a given colony (Table 2 and Table 3). This would be problematic in many cases but obtaining frass samples from a live infestation just prior to fumigation, which have been stored where they could continue to age, could be achieved. This would then allow subsequent comparisons with newly found frass samples post-fumigation to assess if similar aging can be seen and thereby determine if the frass is of newer origin.

### 3.2. Chemical Analysis

Eighteen compounds were identified by GC-MS analysis of the frass extracts. Although their presence and relative concentration differed between samples (Table 4), these compounds provided the best targets for comparison due to their persistence. Many compounds increased or decreased in mean relative percentage area as the frass aged, suggesting that distinctions might be made between classes, although only seven had statistically significant differences between age classes (Table 4). These chemicals were identified as tetradecanal, nonadecane/eicosane (unclear identification within the software), pentadecanal, 3-ethyltetracosane, hexacosane, an unknown oxygenated hydrocarbon, and octacosane. All these compounds were revealed via pairwise tests to be discernable between fresh and old frass (with medium frass being grouped with one or the other) except for tetradecanal, for which fresh and medium frass were shown to be significantly different.

**Figure 3:**
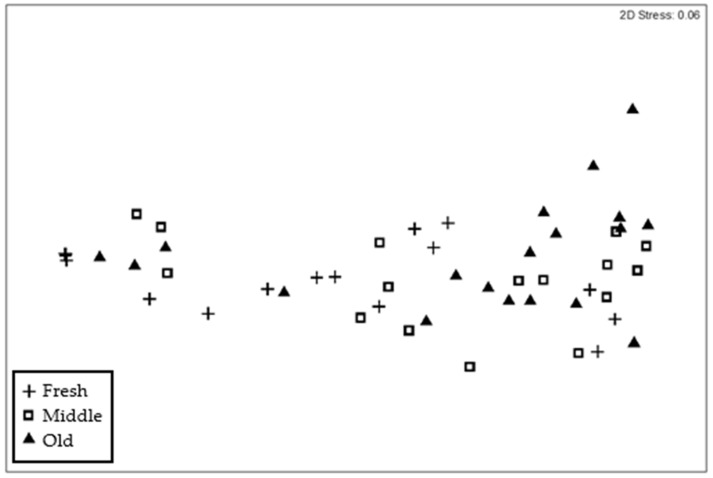
Non-metric multidimensional scaling ordination, showing the distribution of samples from *Cryptotermes brevis* frass. Each point in the ordination represents an individual sample, with age classes demonstrating significant differences, with overlap. Fresh samples are shown with a plus symbol, medium-aged samples are open squares, and old samples are shown as filled triangles.

The nMDS plot (Figure 3) shows that some differences exist between frass age classes, although there was a fair amount of overlap in the data. The analysis of similarity confirmed significant differences between the age groups (global R = 0.067, *p* = 0.047). Pairwise tests showed that only the fresh frass differed significantly from old frass, with no difference between old and medium-aged frass, nor between fresh and medium-aged frass. A similarity percentages (SIMPER) analysis determined that seven compounds accounted for at least 70% of these differences (Table 5) and, notably, just four compounds (heptacosane, pentacosane, steroid 2 and steroid 1) contributed more than 50% of this difference.

## 4. Discussion

The frass color analysis appeared promising at first glance; however, further investigations revealed a critical issue with the technique. The data preliminarily suggested that frass samples start to darken over time, with a longer aging period resulting in increased darkening. This would lend support to the idea of measuring the darkness of a sample as a way to assess its age. However, when these changes were put into context, the initial colors of the samples varied much more than in age-darkened samples by factors of 2 to 19, even when the termites had been fed on an identical food source. This suggests that other factors affect frass color, which varies between similar samples much more notably than with any changes due to aging. Thus, color is not a useful characteristic on which to rely for aging frass. Due to this variability, the color analysis trial was concluded after approximately four months to focus on the chemical analysis methods.

The results of the chemical analysis support the idea of using this technique on *C. brevis* frass as a method of aging the samples. The chemical signatures of frass extracts change significantly as they age, specifically with older frass (12+ months), which presents noticeable differences from fresh frass. This finding agrees with previous work [21], suggesting that changes were only detectable after an extended period of aging. There is no statistically distinguishable difference between fresh frass and the medium-age class of frass. This means that the technique has limits, only being able to distinguish frass that is older than 12 months from fresh frass. Management strategies using this technique will need to account for this issue when determining when to sample the frass. It is important to note that effective fumigation would eliminate active infestations, and there would be a time delay between any re-infestation. Thus, frass from previous infestations is likely to have plentiful time to age before subsequent testing occurs, and the discerned differences in chemistry would be noticeable.

Limitations to the methodologies used are noted, although efforts were made to counter these; most notably, the understanding that the age of the wood veneers used will affect the feeding of the termites and may influence the chemical constituents of the frass produced. By this logic, the veneers used during the trial will also age, and the effect seen in the chemical analysis may be attributed to such changes. However, it is understood that this noted effect on termite feeding is primarily seen in freshly cut wood samples that have not been allowed to age and that contain higher secondary extractive contents. Therefore, wood that had been aged for several years was selected when designing the trial to prevent this occurrence. Additionally, the age of the wood diet has been proposed to affect the gut microbial communities of the termites, which, in turn, may affect the fecal contents. However, a recent work has shown that the core microbial taxa are not altered by the age of the wood consumed [23]. For this reason, the selected methodologies were deemed appropriate for the current study.

For a real-world application of these results, the most important finding is the identification of the key chemical compounds that change in the frass’s chemical profile as it ages. It is these chemicals that could be targeted in the development of a simplified method of sampling the compounds in frass deposits. In its current form, the techniques investigated here have no pragmatic implementation as this analytical approach is generally inaccessible to most homeowners and pest inspectors. Further work is warranted to develop a simple assay that can be deployed in the field to measure the abundance of the key compounds highlighted here (namely, nonadecane/eicosane, pentadecanal, 3-ethyltetracosane, hexacosane, the identified oxygenated hydrocarbon, and octacosane) and produce a resulting age class for the sample. Rigorous field testing of any such tool to confirm its efficacy would provide homeowners and pest inspectors with an easy way to age a discovered frass sample. Additional investigations would be needed to assess if the changes in chemical signatures found here would be seen in a real-world setting in which the frass samples may have spent an extended time aging within the galleries before expulsion, potentially altering the aging patterns found in this study. Current work is ongoing in the use of modern techniques for the identification of *C. brevis*, including DNA assays [24], so the ideas generated here could also be integrated with modern techniques. With influences on termite propagation increasing, such as globalization, intensifying urbanization, and global warming [25], the need for better management of these pests has become more pressing in many countries throughout the world [26,27].

The work described here affords evidence that if the chemical technique suggested by this study is investigated further, this method of determining the approximate age of a termite frass sample may serve as a suitable solution to the specific problem of identifying newly found frass deposits after a building’s treatment. It is hoped that the development of these ideas will lead to an eventual solution that prevents unnecessary fumigation in locations where *C. brevis* is no longer present and can help the afflicted individuals avoid costs and disturbance from this devastating timber pest.

## Figures and Tables

**Table 1:** Age amounts of *C. brevis* frass collected from three colonies and aged for up to 690 days prior to GC-MS analysis.

Age Class	Age Range (Days)	Frass Mass (mg)		
		Colony 1	Colony 2	Colony 3
Fresh	0–39	28.8	48.2	28.1
	40–66	17.8	54.5	25.3
	67–105	16.7	36.2	2.00
	106–133	23.3	0.20	2.60
	134–165	11.0	3.90	5.60
	166–196	41.1	47.9	2.10
Medium	197–228	54.1	19.9	21.9
	229–259	30.7	4.60	19.1
	260–288	110.3	12.3	11.9
	289–312	10.9	36.0	5.80
	313–353	31.2	239.3	70.1
	354–381	213.3	3.90	85.0
Old	382–416	68.4	3.20	25.2
	417–442	50.8	2.90	5.40
	443–499	35.6	14.4	228.2
	500–533	53.7	42.4	48.1
	534–567	56.8	62.9	139.7
	568–599	30.6	31.8	119.9
	600–690	255.5	183.3	453.3

**Table 2:** Effect of *C. brevis* frass age on L*, a*, and b* in the CIELAB color space.

Colony	Sample	Days Aged	L*	a*	b*
Colony 1	Fresh *	0	31.85	19.24	27.62
	Aged	102	24.92	19.91	26.38
Colony 2	Fresh *	0	34.98	7.21	18.42
	Aged	84	34.46	5.66	17.52
Colony 3	Fresh *	0	9.17	2.38	5.08
	Aged	84	7.27	1.86	3.96

* Samples independent of those used for chemical analysis.

**Table 3:** Changes in the color of *C. brevis* frass stored for varying durations, measured as the Euclidian distance (∆E*) between samples in the CIELAB color space. Values greater than 2.3 are considered to have a “just noticeable difference”.

Sample 1	Sample 2	∆E*
Within samples, across time		
Colony 1, Day 0	Colony 1, Day 102	7.068
Colony 2, Day 0	Colony 2, Day 84	1.857
Colony 3, Day 0	Colony 3, Day 84	2.271
Between samples		
Colony 1, Day 0	Colony 2, Day 0	15.469
Colony 1, Day 0	Colony 3, Day 0	36.142
Colony 2, Day 0	Colony 3, Day 0	29.443

**Table 4:** Mean ± SEM of the relative percentage area of compounds that were tentatively identified in extracts of *C. brevis* frass (and the percentage of samples containing the compound) across three age classes: fresh, 0–6 months; medium, 6–12 months; old, 12+ months. Within a row, concentrations followed by the same letter were not statistically distinguishable, with no letters indicating that there were no significant distinctions between any of the classes.

Ret. Time (min)	Compound	Fresh (0–6 Months), 16 Results	Medium (6–12 Months), 19 Results	Old (12+ Months), 19 Results	Friedman
6.55	tetradecanal (C**14**-al)	0.04 ± 0.04 (5) a	0.32 ± 0.12 (47) b	0.16 ± 0.06 (32) ab	Χ^2^_2_ = 7.32, *p* = 0.026
10.05	heptadecanal (C**16**-al)	0.02 ± 0.02 (5)	0.05 ± 0.03 (16)	0.04 ± 0.03 (16)	Χ^2^_2_ = 1.14, n.d.
17.13	nonadecane or eicosane	0.02 ± 0.02 (5) a	0.24 ± 0.10 (32) ab	0.35 ± 0.11 (42) b	Χ^2^_2_ = 6.33, *p* = 0.042
17.79	pentadecanal (C**15**-al)	0 (0) a	0.03 ± 0.03 (5) b	0.07 ± 0.03 (26) b	Χ^2^_2_ = 6.5, *p* = 0.039
18.48	7-hexyldocosane	0.61 ± 0.21 (32)	1.56 ± 0.50 (47)	2.73 ± 0.63 (74)	Χ^2^_2_ = 3.93, n.d.
19.32	pentacosane (C**25**)	14.25 ± 3.06 (58)	20.03 ± 2.92 (68)	19.17 ± 2.69 (79)	Χ^2^_2_ = 2.61, n.d.
20.92	3-ethyltetracosane	2.16 ± 0.65 (47) a	3.62 ± 0.74 (68) ab	5.73 ± 0.88 (79) b	Χ^2^_2_ = 7.65, *p* = 0.022
21.50	hexacosane (C**26**)	0.58 ± 0.31 (21) a	1.90 ± 0.34 (63) b	2.31 ± 0.35 (79) b	Χ^2^_2_ = 10.58, *p* = 0.005
22.27	oxygenated hydrocarbon	0 (0) a	0.21 ± 0.09 (37) ab	0.41 ± 0.24 (42) b	Χ^2^_2_ = 9.25, *p* = 0.01
23.73	heptacosane (C**27**)	21.59 ± 4.40 (63)	29.54 ± 3.10 (84)	29.74 ± 2.93 (100)	Χ^2^_2_ = 4.63, n.d.
25.31	9-octylhexacosane	0.12 ± 0.07 (16)	0.37 ± 0.10 (42)	0.31 ± 0.07 (53)	Χ^2^_2_ = 5.78, n.d.
28.06	octacosane (C**28**)	0.46 ± 0.24 (21) a	1.44 ± 0.26 (63) b	1.71 ± 0.28 (74) b	Χ^2^_2_ = 10.33, *p* = 0.012
32.37	cholesterol	0.20 ± 0.11 (16)	0.20 ± 0.09 (37)	0.11 ± 0.09 (16)	Χ^2^_2_ = 2.44, n.d.
32.54	cholestanol	0.13 ± 0.10 (11)	0.34 ± 0.13 (37)	0.26 ± 0.11 (42)	Χ^2^_2_ = 3.21, n.d.
33.77	steroid 1	22.91 ± 3.11 (84)	17.08 ± 3.84 (84)	14.61 ± 2.98 (95)	Χ^2^_2_ = 3.88, n.d.
36.56	stigmastanol	0.27 ± 0.13 (21)	0.65 ± 0.28 (42)	1.00 ± 0.31 (79)	Χ^2^_2_ = 5.65, n.d.
37.55	steroid 2	25.66 ± 3.89 (84)	14.17 ± 2.44 (84)	14.24 ± 2.64 (100)	Χ^2^_2_ = 3.50, n.d.
42.07	steroid 3	10.90 ± 1.87 (84)	8.06 ± 1.50 (84)	6.68 ± 1.18 (100)	Χ^2^_2_ = 2.62, n.d.

**Table 5:** The most frequently identified compounds from *Cryptotermes brevis* frass extracts, accounting for at least 70% of the differences between age-class samples.

Compound	% Contribution to Dissimilarity ^1^	Cumulative %
heptacosane (C**27**)	14.24	14.24
pentacosane (C**25**)	13.79	28.03
steroid 2	11.40	39.43
steroid 1	11.21	50.64
3-ethyltetracosane	8.49	59.13
steroid 3	7.83	66.97
hexacosane (C**26**)	6.34	73.31

^1^ SIMPER-identified value, indicating how the compound deviated in the samples from the mean values across all data.

## Data Availability

The raw data supporting the conclusions of this article will be made available by the authors upon request.
